# Scoring Health Behaviors of Patients with Type 2 Diabetes

**DOI:** 10.3390/medicina60101644

**Published:** 2024-10-08

**Authors:** Aleksandra Lidia Jaworska-Czerwińska, Katarzyna Oliwa-Libumska, Marta Lewicka, Przemysław Żuratyński

**Affiliations:** 1 Department of Emergency Medical Services, Faculty of Health Sciences, Collegium Medicum in Bydgoszcz, Nicolaus Copernicus University in Torun, 85-094 Bydgoszcz, Poland; aleksandra.czerwinska@cm.umk.pl; 2 Department of Anesthesiology and Intensive Care for Adults, 10th Military Teaching Hospital with Polyclinic SPZOZ, 85-681 Bydgoszcz, Poland; katarzyna.oliwa@wp.pl; 3 Department of Preventive Nursing, Faculty of Health Sciences, Collegium Medicum in Bydgoszcz, Nicolaus Copernicus University in Torun, 85-094 Bydgoszcz, Poland; marta.lewicka@cm.umk.pl

**Keywords:** patient, lifestyle, diabetes, HLPCQ

## Abstract

*Background and Objectives*: Millions of people worldwide suffer from diabetes. The ever-increasing number of patients poses a huge challenge to healthcare systems. The purpose of this study was to evaluate the lifestyle and self-monitoring of type 2 diabetes patients using the Healthy Lifestyle and Self-Monitoring Questionnaire. *Material and Methods*: The analyses conducted were based on data collected using the Polish version of the Healthy Lifestyle and Self-Control Questionnaire among 104 patients diagnosed with type 2 diabetes who were treated at the Diabetes Outpatient Clinic. The in-house study also included an analysis of the relationship between lifestyle habits and disease acceptance and chronic disease functioning. *Results*: Respondents scored statistically significantly higher for the Healthy Lifestyle and Self-Monitoring Questionnaire than the norms assume, and the largest differences were observed in terms of the healthy dietary choices subscale (t = 8.07; *p* < 0.05). Only for the subscale of organized exercise were no statistically significant differences found (t = 0.50; *p* = 0.620). *Conclusions*: Type 2 diabetes is one of the diseases in which lifestyle not only contributes to its development but is also associated with its course and treatment outcomes. Reinforcing a health-promoting lifestyle is one of the cornerstones of treating patients with type 2 diabetes.

## 1. Introduction

Globally, the number of people with diabetes is increasing at a rapid pace. It is estimated that one in eleven adults is diagnosed with diabetes. In Poland, the disease affects one in four people over the age of 60 [1]. It is a chronic disease that can eventually lead to many complications. The World Health Organization predicts that diabetes will be the seventh leading cause of death worldwide by 2030 [1]. According to these projections, it will be a huge challenge not only for medical personnel but also for entire healthcare systems.

Diabetes contributes to the existence of many difficulties in daily life and inconveniences due to factors associated with the disease. It is also reflected in the emotional and psychological functioning of patients and is a major source of stress, leading to a deterioration in patients’ quality of life [2].

According to Lalonde’s concept, a person’s health is most affected by lifestyle (50%). It is also the only factor that depends on the individual. The other factors—biological, environmental, and healthcare—do not have as much influence. According to the definition, lifestyle is “a set of ordinary daily decisions, activities, habits and actions characteristic of an individual [3]. This individual approach may be subject to modification due to changes in a person’s environment, changes in attitude, financial resources, beliefs, past illnesses, and experience [3]. Interest in the lifestyle and quality of life of patients and their functioning in illness is embedded in the accepted model of holistic care [4,5].

The aim of the study was to assess the lifestyle and self-care of patients with type 2 diabetes using the Healthy Lifestyle and Personal Control Questionnaire (HLPCQ). The obtained results were also analyzed for the presence of a relationship between lifestyle, disease acceptance, and functioning with a chronic illness, which were also examined by the authors using reliable research tools. A previous literature review indicates that the number of studies utilizing the HLPCQ is limited, which justifies the conduct of this research. Furthermore, the obtained results may provide valuable insights into patients’ health behaviors and their impact on the course of treatment and readiness to change their lifestyle.

## 2. Materials and Methods

Our study was conducted on the basis of the Polish version of the Healthy Lifestyle and Personal Control Questionnaire (HLPCQ), published in 2021 by M. Czapla et al. [6]. The study was conducted between July and November 2023 among patients of the Diabetology Clinic at Jan Biziel University Hospital in Bydgoszcz. During this period, all patients receiving treatment for type 2 diabetes were informed by the nurses working at the Clinic about the opportunity to participate in the study. A total of 104 patients volunteered, all of whom were included in the study. The study was conducted in accordance with ethical principles, the source of which is the Declaration of Helsinki. Approval was obtained from the Bioethics Committee at the Nicolaus Copernicus University in Torun, Collegium Medicum in Bydgoszcz (KB 249/2023).

The HLPCQ questionnaire consists of 26 statements—positively rated lifestyle habits. Respondents were asked to indicate the frequency of their display using a Likert-type scale (1 = rarely or never, 2 = sometimes, 3 = often, 4 = always). Individual statements were assigned to one of five subscales: Healthy Dietary Choices, Dietary Harm Avoidance, Daily Routine, Structured Exercise, and Social Support and Mental Health. An overall assessment was made, as well as an assessment for each of the subscales. The internal consistency of the HLPCQ, measured by Cronbach’s alpha coefficient, is 0.898

The in-house research also included an analysis of the relationship between lifestyle habits and disease acceptance and chronic disease functioning. In this part of the study, the Acceptance of Illness Scale (AIS) in the Polish adaptation by Z. Juczynski [7] and The Functioning in the Chronic Illness Scale (FCIS) by A. Kubica [8] were used.

### 2.1. Characteristics of the Study Population

A total of 104 people were surveyed, including 68 women, accounting for 65.4% of the respondents, and 36 men, accounting for 34.6%. All participants were diagnosed with type 2 diabetes. The average age of the respondents was 66.45 (±10.37), and the largest number of respondents were in the 61–70 age range (42.3%). The largest percentage of respondents (44.3%), 46 people, had secondary education, while vocational education was held by 23 people (22.1%), and higher education was held by 24 people (23.1%). In terms of the source of livelihood, the most numerous, 68 people in the study group, were people receiving a pension (66.45%), while for 23 respondents, the source of livelihood was a job (22.1%), for 10 people, it was a pension (9.6%), 2 people, while retired, additionally engaged in casual work (1.9%), and 1 person was studying. Among the surveyed patients, those with diabetes diagnosed within 11–20 years (33.7%) from the date of the survey predominated. Next in terms of numbers were those diagnosed up to 5 years (26%) and within 6–10 years (16.3%) from the date of the survey. In 2023, 4.8% of people were diagnosed with diabetes, 72.1% had comorbidities, and 50.0% were diagnosed with hypertension. There was incidence of diabetes in the relatives of 43.3% of the respondents—the most common being the parents of the respondents, while less common were the children of the respondents, siblings, grandparents, or extended family members. Subcutaneous insulin injections were performed in 33 respondents (31.7%).

### 2.2. Statistical Analyses

Significance tests for differences and correlation coefficients were used in the analyses. The normality of the distribution of the data collected from respondents was checked using the Shapiro–Wilk W test. Differences in two populations in terms of a given quantitative variable were assessed using the Mann–Whitney U test. Spearman’s rank correlation was used to test the correlation of two variables that did not meet the criterion of normality of distribution and variables of ordinal nature. Pearson’s r correlations were used for quantitative variables with a normal distribution. A one-sample Student’s *t* test was used to test whether the level of acceptance of the disease corresponds to the norm for diabetics. IBM SPSS Statistic 23 was used to analyze the collected data. *p* < 0.05 was used as the level of statistical significance.

## 3. Results

### 3.1. Statistical Analysis of the HLPCQ Questionnaire

The subjects assessed their attitudes toward healthy lifestyle and self-control using the HLPCQ questionnaire (Table 1). The scatter of the obtained results ranged from 26 to 94 points with a mean of 70.13 points and a deviation of ±12.90 points. On the healthy dietary choices subscale, the subjects scored between 7 and 26 points with a mean of 17.82 points and a variance of ±4.08 points, and on the dietary harm avoidance subscale, the subjects scored between 4 and 16 points with a mean of 12.04 points and a variance of ±2.83 points. Then, on the daily routine subscale, the subjects scored between 8 and 32 points with a mean of 23.33 points and a variance of ±5.16 points, and on the organized exercise subscale, they scored between 2 and 8 points with a mean of 4.21 points and a variance of ±1.56 points. The level of social support and mental health in the study group was between 5 and 20 points, with a mean of 12.73 points and a deviation of ±3.25 points.

The results of normality analyses conducted with the Shapiro–Wilk test showed that the distribution of the healthy lifestyle scale and self-control scale differed slightly from the normal distribution *p* < 0.05, but the scales did not have strongly skewed distributions. The subjects obtained a high reliability of questionnaire responses, with α > 0.7. The results of the analyses are shown in Table 1.

Next, the level of the Healthy Lifestyle Scale and self-monitoring were compared with the assumed norms. For this purpose, a comparative analysis was performed using Student’s *t* test for one sample. The results of these analyses are shown in Table 2. Based on them, it can be concluded that the healthy lifestyle and self-control scales were statistically significantly different *p* < 0.05 from the assumed norms, with the exception of the subscale of organized exercise (t = 0.50; *p* = 0.620). The subjects with type 2 diabetes scored statistically significantly higher on the healthy lifestyle and self-monitoring scales than the assumed norms, with the largest differences shown for the healthy dietary choices subscale.

We also examined how gender, age, and education are associated with HLPCQ questionnaire scores. Table 3 shows the results of Spearman’s rho correlation analyses for the association of the healthy lifestyle and self-monitoring scales with age and education. The results of these analyses were mostly found to be statistically insignificant. A statistically significant association was only found between the age of the subjects and the daily routine subscale, with rho = 0.19; *p* < 0.05. This association was positive, meaning that older subjects were more likely to submit to a daily routine and daily plan. A series of Spearman’s rho correlation analyses also showed no statistically significant association between education and the severity of the Healthy Lifestyle and Self-Management Scale (rho = −0.05; *p* = 0.629) and its subscales in the type 2 diabetes study group.

In turn, by means of a series of analyses performed with Mann–Whitney U tests, we examined whether the gender of the subjects differentiated their variation in the healthy lifestyle and self-control scales. Based on the results shown in Table 4, it can be concluded that gender differentiated the level of the healthy lifestyle and self-control scale (Z = 1.96; *p* = 0.050; r = 0.19 (a result at the limit of statistical tendency)) and the level of the healthy food choices subscale (Z = 2.74; *p* < 0.01; r = 0.27). In the study group, women had higher levels of healthy lifestyle and self-control and healthy dietary choices. There were no differences between men and women in terms of the other subscales of healthy lifestyle and self-control.

### 3.2. Relationship of Disease Acceptance with Healthy Lifestyle and Self-Control

The purpose of the study was to analyze the relationship of disease acceptance and healthy lifestyle and self-control. For this purpose, Pearson’s r correlation analysis was performed, and the results are shown in Table 5. The results showed that there was a statistically significant association of disease acceptance with healthy lifestyle and self-control (r = 0.36; *p* < 0.001), as well as with dietary harm avoidance (r = 0.22; *p* < 0.05), daily routine (r = 0.32; *p* < 0.01), structured physical exercise (r = 0.25; *p* < 0.05), and social support and mental health (r = 0.35; *p* < 0.01). These associations were positive, meaning that those with high levels of disease acceptance had high levels of healthy lifestyle and self-control. Only a statistically significant association of disease acceptance with the healthy dietary choices scale was not shown (r = 0.18; *p* = 0.087).

### 3.3. Level of Functioning in Illness versus Healthy Lifestyle and Self-Management

The level of functioning was assessed using the FCIS questionnaire, which consists of three subscales and assesses the impact of the disease on the patient, the impact of the patient on the disease, and the impact of the disease on the patient’s attitudes. The sum of these subscales is the score, where a maximum score means that the patient functions very well with the disease while a minimum value means that the patient functions poorly with the disease. Using Pearson’s r correlation analyses, the relationship between people’s functioning in illness and healthy lifestyle and self-management was examined. The results of these analyses are shown in Table 6. Based on these analyses, it can be concluded that the overall illness impact scale was statistically significantly associated with the social support and mental health subscale (r = 0.21; *p* < 0.05), and the illness impact scale was statistically significantly associated with the organized exercise subscale (r = 0.25; *p* < 0.05). It was also found that high levels of patient impact on illness attitudes were statistically significantly associated with the healthy lifestyle and self-control scale (r = 0.30; *p* < 0.01) and the subscales of dietary harm avoidance (r = 0.22; *p* < 0.05), organized exercise (r = 0.25; *p* < 0.05), and social support and mental health (r = 0.33; *p* < 0.01). The overall scale of patient functioning in illness was statistically significantly associated with the healthy lifestyle and self-control scale (r = 0.26; *p* < 0.05), and with the subscales of organized exercise (r = 0.27; *p* < 0.01) and social support and mental health (r = 0.27; *p* < 0.01). These associations were positive, meaning that people with diabetes characterized by high functioning in illness also had higher levels of the healthy lifestyle and self-control scales. The strongest associations were shown with the social support and mental health scales, which may mean that the support of loved ones and not only physical but also mental health are important in maintaining health.

## 4. Discussion

According to numerous studies, a health-enhancing lifestyle can help prevent almost 90% of cases of type 2 diabetes, more than 80% of cardiovascular diseases, 50% of strokes, and 30% of cancers [6,9,10].

The studies presented here show that patients with type 2 diabetes generally score better on the HLPCQ questionnaire, meaning that they engage in more health-enhancing behaviors than the general population. Lifestyle is a very important aspect of non-pharmacological treatment, including normalization of blood glucose levels.

The patients surveyed paid the most attention to making healthy dietary choices. In this part of the survey, respondents had several statements to choose from regarding the following issues: paying attention to the amount of food on the plate, checking the labels of the products purchased, counting calories in meals, limiting the number of fats in meals, and eating whole grains and organic (organic) products. One statement asked whether respondents liked to cook. Published in 2023, a review by T.P. Minari et al. of 202 randomized clinical trials, systematic reviews, meta-analyses, and guidelines from 1983–2023 showed that a Mediterranean diet may be more important in the management of type 2 diabetes with the following dietary recommendation: 40–50% carbohydrate, 15–25% protein, 25–35% fat (<7% saturated, 10% polyunsaturated and 10% monounsaturated), at least 14 g of fiber for every 1000 calories consumed, and <2300 mg of sodium. The analyses also highlighted the importance of individualization as the gold standard for dietary recommendations in this group of patients [11]. In the studied group, women exhibited higher levels of healthy lifestyle and self-control, as well as healthier dietary choices. No differences were observed between women and men regarding the other subscales of healthy lifestyle and self-control. In Poland, women are more likely than men to purchase food products and typically make the decisions about the food items bought for the household. Additionally, women are more inclined than men to choose lower-calorie foods, strive for optimal nutrition, and follow trends in healthy eating, motivated by concerns about weight control and their more frequent engagement in dieting [12].

In our own study, our respondents scored lower on the structured exercise subscale. This part of the questionnaire included statements about regular aerobic exercise for 20 min or more a minimum of three times a week and a question about exercising/training according to a structured program. Physical activity is essential in the fight against overweight and obesity. Reducing body fat improves tissue sensitivity to insulin, reducing insulin resistance. Visceral obesity is a modifiable risk factor for type 2 diabetes [13]. Despite such obvious evidence, not all patients engage in regular physical activity—the most common form of activity is walking [14,15]. Lifestyle interventions, including weight loss and physical activity, are important aspects in the management of type 2 diabetes and the prevention of complications associated with atherosclerotic cardiovascular disease. Weight loss and physical activity are widely promoted as the first line of treatment to prevent many of the chronic complications associated with diabetes. Higher levels of physical activity and cardiorespiratory fitness are associated with a lower risk of mortality and adverse cardiovascular events among patients with type 2 diabetes, as well as preventing weight regain, improving glycemic control and quality of life, and reducing the risk of heart failure [16].

Our own research also showed that people with diabetes characterized by a high level of acceptance of the disease and a high level of functioning in the disease had higher levels of healthy lifestyle and self-control. This is a very important aspect, as adopting a positive attitude facilitates cooperation with doctors and determines appropriate decision-making [17,18]. The literature emphasizes the importance of good quality of life—in addition to striving to achieve target values of glycemia, blood pressure, lipidogram, or body weight—as a guideline for therapeutic teams responsible for the treatment of patients with type 2 diabetes [19]. Measuring quality of life and identifying prognostic factors provide insight into patients’ needs and the establishment of evidence-based targeted prevention programs, including education and support, and may prove helpful in treatment planning [20,21,22,23]. It seems necessary to conduct more extensive studies of this type, involving numerous diabetology clinics across Poland, located in various regions. This would allow for the exclusion of the influence of local healthcare service quality and availability (small towns vs. large urban areas). Moreover, the use of behavioral models, such as the health belief model, could prove helpful in assessing the determinants of differences in healthy lifestyles and self-management. This would aid in explaining individuals’ decisions regarding health-related behaviors. Obtaining a comprehensive picture may contribute to reducing the risk of complications and improving the treatment of patients with type 2 diabetes [24].

### Limitations of the Work

Due to the prevalence of type 2 diabetes, one of the limitations of the study is the relatively small number of subjects studied. In addition, the study was conducted in only one diabetes clinic. Data were collected on the basis of a standardized questionnaire, the completion of which was voluntary and anonymous—for this reason, it was not possible to relate the results obtained to the clinical data and current health status of the patients.

## 5. Conclusions

The study shows that the HLPCQ questionnaire can be a useful tool for assessing the lifestyle patterns of patients with type 2 diabetes, which provide an opportunity to reflect health empowerment and internal locus of control over health. The tool used was characterized by usefulness, reliability, and consistency. The surveyed patients diagnosed with type 2 diabetes scored higher than the assumed norms, indicating a higher level of healthy lifestyle and self-control than the general population. Our own research also shows that people with diabetes pay more attention to making healthy dietary choices. The level of physical activity remains inadequate among those surveyed.

## Figures and Tables

**Table 1:** Descriptive statistics for healthy lifestyle and self-monitoring scales.

	Min	Max	M	SD	Me	Sk	K	*p*	α
Healthy lifestyle and self-control	26	94	70.13	12.90	70	–0.60	0.49	0.042	0.89
Healthy dietary choices	7	26	17.82	4.08	18	–0.13	–0.29	0.324	0.74
Dietary harm avoidance	4	16	12.04	2.83	12	–0.57	–0.08	0.001	0.61
Daily routine	8	32	23.33	5.16	23	–0.44	–0.09	0.015	0.85
Organized physical exercise	2	8	4.21	1.56	4	0.34	–0.58	<0.001	0.61
Social support and mental health	5	20	12.73	3.25	13	–0.15	–0.48	0.112	0.74

Min—minimum value, Max—maximum value, M—mean, SD—standard deviation, Me—median, Sk—skewness, K—kurtosis, *p*—significance by Shapiro–Wilk test, α—Cronbach’s alpha.

**Table 2:** Comparison of healthy lifestyle and self-monitoring scales with the norm.

	M	SD	Mt	t	*p*
Healthy lifestyle and self-control	70.13	12.90	60.96	7.21	<0.001
Healthy dietary choices	17.82	4.08	14.57	8.07	<0.001
Dietary harm avoidance	12.04	2.83	10.39	5.91	<0.001
Daily routine	23.33	5.16	19.82	6.90	<0.001
Organized physical exercise	4.21	1.56	4.29	0.50	0.620
Social support and mental health	12.73	3.25	11.87	2.69	0.009

M—mean, SD—standard deviation, Mt—theoretical mean, t—test result, *p*—level of statistical significance.

**Table 3:** Results of Spearman’s rho correlation analyses for the association of the healthy lifestyle and self-monitoring scale with age and education.

	rho/*p*	Age	rho/*p*	Education
Healthy lifestyle and self-control	rho	0.10	rho	–0.05
	*p*	0.312	*p*	0.629
Healthy dietary choices	rho	<0.01	rho	–0.09
	*p*	0.997	*p*	0.358
Dietary harm avoidance	rho	0.07	rho	0.06
	*p*	0.480	*p*	0.541
Daily routine	rho	0.19	rho	0.03
	*p*	0.049	*p*	0.788
Organized physical exercise	rho	–0.08	rho	–0.06
	*p*	0.433	*p*	0.528
Social support and mental health	rho	0.08	rho	–0.07
	*p*	0.403	*p*	0.493

rho—Spearman’s rho statistic, *p*—level of statistical significance.

**Table 4:** Result of analysis by Mann–Whitney U test for comparison of healthy lifestyle and self-control scales in disease by gender.

	Women		Men		Z	*p*	r
	M	SD	M	SD			
Healthy lifestyle and self-control	71.60	14.05	67.39	10.05	1.96	0.050	0.19
Healthy dietary choices	18.57	4.28	16.42	3.30	2.74	0.006	0.27
Dietary harm avoidance	12.09	2.98	11.94	2.56	0.52	0.601	0.05
Daily routine	23.66	5.45	22.72	4.60	1.24	0.216	0.12
Organized physical exercise	4.24	1.59	4.17	1.52	0.14	0.888	0.01
Social support and mental health	13.04	3.51	12.14	2.65	1.43	0.154	0.14

M—mean, SD—standard deviation, Z—Mann–Whitney U statistic, *p*—level of statistical significance, r—magnitude of differences.

**Table 5:** Results of Pearson’s r correlation analyses for the association of the disease acceptance scale and the healthy lifestyle and self-control scale.

	r/*p*	Acceptance of the Disease
Healthy lifestyle and self-control	r	0.36
	*p*	<0.001
Healthy dietary choices	r	0.18
	*p*	0.087
Dietary harm avoidance	r	0.22
	*p*	0.038
Daily routine	r	0.32
	*p*	0.002
Organized physical exercise	r	0.25
	*p*	0.014
Social support and mental health	r	0.35
	*p*	0.001

r—magnitude of differences, *p*—level of statistical significance.

**Table 6:** Results of Pearson’s r correlation analyses for the association of the disease impact scale and the healthy lifestyle and self-control scale.

	r/*p*	The Impact of the Disease on the Patient	Patient’s Impact on Illness	Patient’s Impact on Patient’s Attitudes	Patient’s Overall Functioning in Illness
Healthy lifestyle and self-control	r	0.18	0.11	0.30	0.26
	*p*	0.085	0.281	0.003	0.011
Healthy dietary choices	r	0.09	0.15	0.20	0.18
	*p*	0.411	0.149	0.054	0.089
Dietary harm avoidance	r	0.09	0.11	0.22	0.18
	*p*	0.390	0.277	0.030	0.085
Daily routine	r	0.14	0.00	0.18	0.16
	*p*	0.171	0.994	0.073	0.132
Organized physical exercise	r	0.17	0.25	0.25	0.27
	*p*	0.100	0.014	0.015	0.008
Social support and mental health	r	0.21	0.03	0.33	0.27
	*p*	0.044	0.748	0.001	0.009

r—magnitude of differences, *p*—level of statistical significance.

## Data Availability

The raw data supporting the conclusions of this article will be made available by the authors on request to the corresponding author.
